# Therapeutic Targeting of Tumor Cells and Tumor Immune Microenvironment Vulnerabilities

**DOI:** 10.3389/fonc.2022.816504

**Published:** 2022-06-08

**Authors:** Balaraman Kalyanaraman, Gang Cheng, Micael Hardy

**Affiliations:** ^1^ Department of Biophysics, Medical College of Wisconsin, Milwaukee, WI, United States; ^2^ Center for Disease Prevention Research, Medical College of Wisconsin, Milwaukee, WI, United States; ^3^ Aix Marseille Univ, Centre National de la Recherche Scientifique (CNRS), Institut de Chimie Radicalaire (ICR), Marseille, France

**Keywords:** Mitochondrial drugs, tumor microenvironment, metabolic reprogramming, oxidative phosphorylation (OXPHOS), monocarboxylate transporters

## Abstract

Therapeutic targeting of tumor vulnerabilities is emerging as a key area of research. This review is focused on exploiting the vulnerabilities of tumor cells and the immune cells in the tumor immune microenvironment (TIME), including tumor hypoxia, tumor acidity, the bidirectional proton-coupled monocarboxylate transporters (MCTs) of lactate, mitochondrial oxidative phosphorylation (OXPHOS), and redox enzymes in the tricarboxylic acid cycle. Cancer cells use glucose for energy even under normoxic conditions. Although cancer cells predominantly rely on glycolysis, many have fully functional mitochondria, suggesting that mitochondria are a vulnerable target organelle in cancer cells. Thus, one key distinction between cancer and normal cell metabolism is metabolic reprogramming. Mitochondria-targeted small molecule inhibitors of OXPHOS inhibit tumor proliferation and growth. Another hallmark of cancer is extracellular acidification due lactate accumulation. Emerging results show that lactate acts as a fuel for mitochondrial metabolism and supports tumor proliferation and growth. Metabolic reprogramming occurs in glycolysis-deficient tumor phenotypes and in kinase-targeted, drug-resistant cancers overexpressing OXPHOS genes. Glycolytic cancer cells located away from the vasculature overexpress MCT4 transporter to prevent overacidification by exporting lactate, and the oxidative cancer cells located near the vasculature express MCT1 transporter to provide energy through incorporation of lactate into the tricarboxylic acid cycle. MCTs are, therefore, a vulnerable target in cancer metabolism. MCT inhibitors exert synthetic lethality in combination with metformin, a weak inhibitor of OXPHOS, in cancer cells. Simultaneously targeting multiple vulnerabilities within mitochondria shows synergistic antiproliferative and antitumor effects. Developing tumor-selective, small molecule inhibitors of OXPHOS with a high therapeutic index is critical to fully exploiting the mitochondrial vulnerabilities. We and others developed small-molecule inhibitors containing triphenylphosphonium cation that potently inhibit OXPHOS in tumor cells and tissues. Factors affecting tumor cell vulnerabilities also impact immune cells in the TIME. Glycolytic tumor cells supply lactate to the tumor-suppressing regulatory T cells overexpressing MCTs. Therapeutic opportunities for targeting vulnerabilities in tumor cells and the TIME, as well as the implications on cancer health disparities and cancer treatment, are addressed.

## Identifying the Vulnerabilities in Cancer Cell Metabolism

Investigators from two leading genomics research institutes—*the Wellcome-Sanger Institute in the United Kingdom* and *the Broad Institute in Cambridge, MA*—started a pilot project in search of a cancer dependency map (1). The objective was to map out the gene, protein, or other molecular feature that a tumor depends on for growth. The cancer dependency is also a cancer vulnerability.

Cancer cells and stromal cells in the tumor immune microenvironment (TIME) are dependent on several factors (nutrients such as glucose, glutamine, fatty acid, molecular oxygen; an acidic environment caused by increased lactate; and monocarboxylate transporter [MCT] proteins, redox control of reactive oxygen species [ROS] generation, and ROS levels in mitochondria) (2–6). One of the key vulnerable target organelles is mitochondria in cancer cells (4, 6–8). Cancer cells use up all the nutrients and oxygen very rapidly such that these cells constantly lack nutrients and oxygen. This makes cancer cells vulnerable to drugs that induce modifications in processes regulating acidity, hypoxia, and nutrient availability. The pKa of the lactate/lactic acid pair is 3.8, and at physiological pHs, it exists as a lactate anion and a hydrogen ion. Because lactate is negatively charged, its transport is facilitated by specific transport proteins in cell membranes. The MCT proteins, a family of transmembrane proteins, mediate the proton-linked bidirectional movement of lactate in and out of cells (9). Cancer cells are vulnerable to modifications in MCTs. The presence of high levels of antioxidant enzymes in cancer cells is attributed to the need to detoxify excessive ROS levels (10). Redox enzymes in the tricarboxylic acid (TCA) cycle (which is also referred to as the Krebs cycle) in mitochondria are vulnerable targets in cancer cells (11). Ferroptosis (iron-induced ROS-mediated apoptosis) is regulated by mitochondrial enzymes, and is a vulnerable target in cancer (12). In addition to vulnerabilities such as decreased extracellular acidification, decreased tumor hypoxia, decreased nutrients, and increased expression of MCT transporters and metabolites, tumor cells are susceptible to modifications of mitochondrial fusion and fission, and to mitochondrial translation (13).

## The Warburg Effect: Aerobic Glycolysis and the Preferred Pathway for Acquiring Energy in Cancer Cells

**Figure 1:**
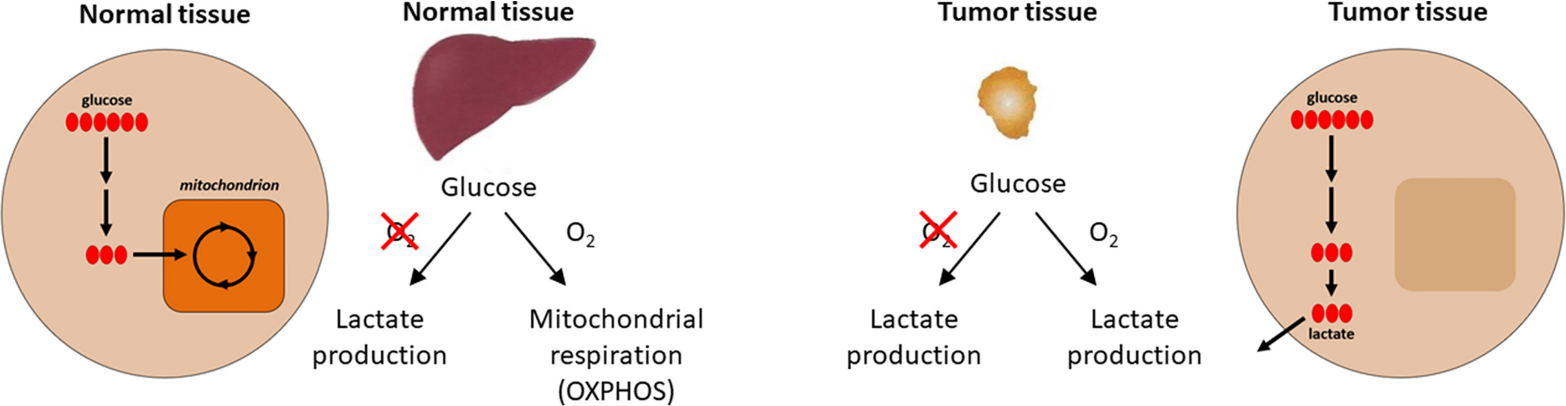
Aerobic metabolic differences between normal and tumor tissues. Under aerobic conditions, normal tissues use OXPHOS for ATP production and cancer cells use glycolysis (the Warburg effect) for energy. Modified from an image created by the Patti Lab, Washington University, that appeared in the October 20, 2016, National Cancer Institute Cancer Currents Blog post, *Metabolomics Study Reveals another Energy Source for Cancer Cells*, with permission from Gary Patti.

One of the first biochemical distinctions noted between cancer tissues and normal tissues was the difference in metabolism or metabolic reprogramming (14, 15). In the 1920s, biochemist Otto H. Warburg observed that in the presence of glucose, cancer tissues generate plenty of lactate, even when oxygen is present (*i.e.*, under normoxic conditions) (16). This is different from most normal tissues that only ferment glucose to lactate in the absence of oxygen or hypoxia. This metabolic reprogramming observed in cancer cells is referred to as the Warburg effect (the name was given by Efraim Racker in 1972) (**Figure 1**). Throughout this article, we use the term “metabolic reprogramming” to indicate the ability of cancer cells to alter their metabolism in order to support their enhanced energy requirements to help sustain the rapid proliferation and growth that are typical hallmarks of tumor cells. This discovery (*i.e*., the Warburg effect) led to the development of cancer diagnostic imaging modality—the PET (positron emission tomography) scan that is used to monitor enhanced glucose uptake in tumor tissues. This imaging modality uses the glucose analog, fluoro-2-deoxy-D-glucose, as a probe where the fluorine-18 atom is a positron-emitting source. Fluoro-2-deoxy-D-glucose is taken up into cancer cells, phosphorylated by hexokinase, and trapped in the cells as it cannot be metabolized further (17). However, to rationalize as to why cancer cells prefer aerobic glycolysis for energy, Warburg hypothesized that cancer cells have defective mitochondria that result in impaired aerobic respiration, thereby forcing their dependency on glycolytic metabolism. This hypothesis was proven to be incorrect in some respects because studies showed that mitochondrial function was not impaired in most cancer cells (18). Furthermore, reprogramming of cancer cell bioenergetics from glycolysis to mitochondrial oxidative metabolism occurs when glycolysis is compromised (19). Recently, using hyperpolarized magnetic resonance imaging, Oshima et al. demonstrated the occurrence of metabolic rewiring between mitochondrial metabolism and glycolysis, and the impact of metabolic targeting of mitochondrial OXPHOS and lactate dehydrogenase on tumor growth (20). OXPHOS and hypoxia share a reciprocal relationship and are potential targets for developing cancer therapies.

## Lactate and MCTs: Fuel for Oxidative Tumor Cells

Using “untargeted metabolomics” approaches (nuclear magnetic resonance [NMR] and liquid chromatography–mass spectrometry), investigators (including graduate students and postdoctoral fellows) tracked down the movement of carbon-13 (^13^C)-labeled lactate and found that nearly all lipids in the cell are a metabolic product of lactate. The investigators ended up tagging the ^13^C-labeled atom from ^13^C lactate (21). This finding was clearly unexpected. Also, it suggests that mitochondria in cancer cells can use lactate to fuel biochemical reactions responsible for cell growth (21). Lactate was shown to be a TCA cycle carbon source for non-small-cell lung carcinoma (22). The infusion of ^13^C lactate in human non-small-cell lung carcinoma patients revealed extensive labeling of TCA cycle metabolites (22). Deleting monocarboxylate transporter 1 (MCT1) from tumor cells eliminated lactate-dependent metabolite labeling. Directly comparing lactate and glucose metabolism *in vivo* revealed that lactate’s contribution to the TCA cycle predominates (23). These results indicate that tumors can use lactate as a fuel *in vivo*. Recently, it was reported that tumor-derived lactate was used as fuel by regulatory T cells (T_regs_) in the TIME (24).

## AZD3965 Blocks the Activity of MCT1

Lactate was discovered as the TCA cycle carbon and energy source for lung tumors in mice (25). The investigators assumed that lactate was metabolized in the cytosol rather than in mitochondria. The reasoning was that if lactate is a waste product, it should not be in mitochondria.

**Figure 2:**
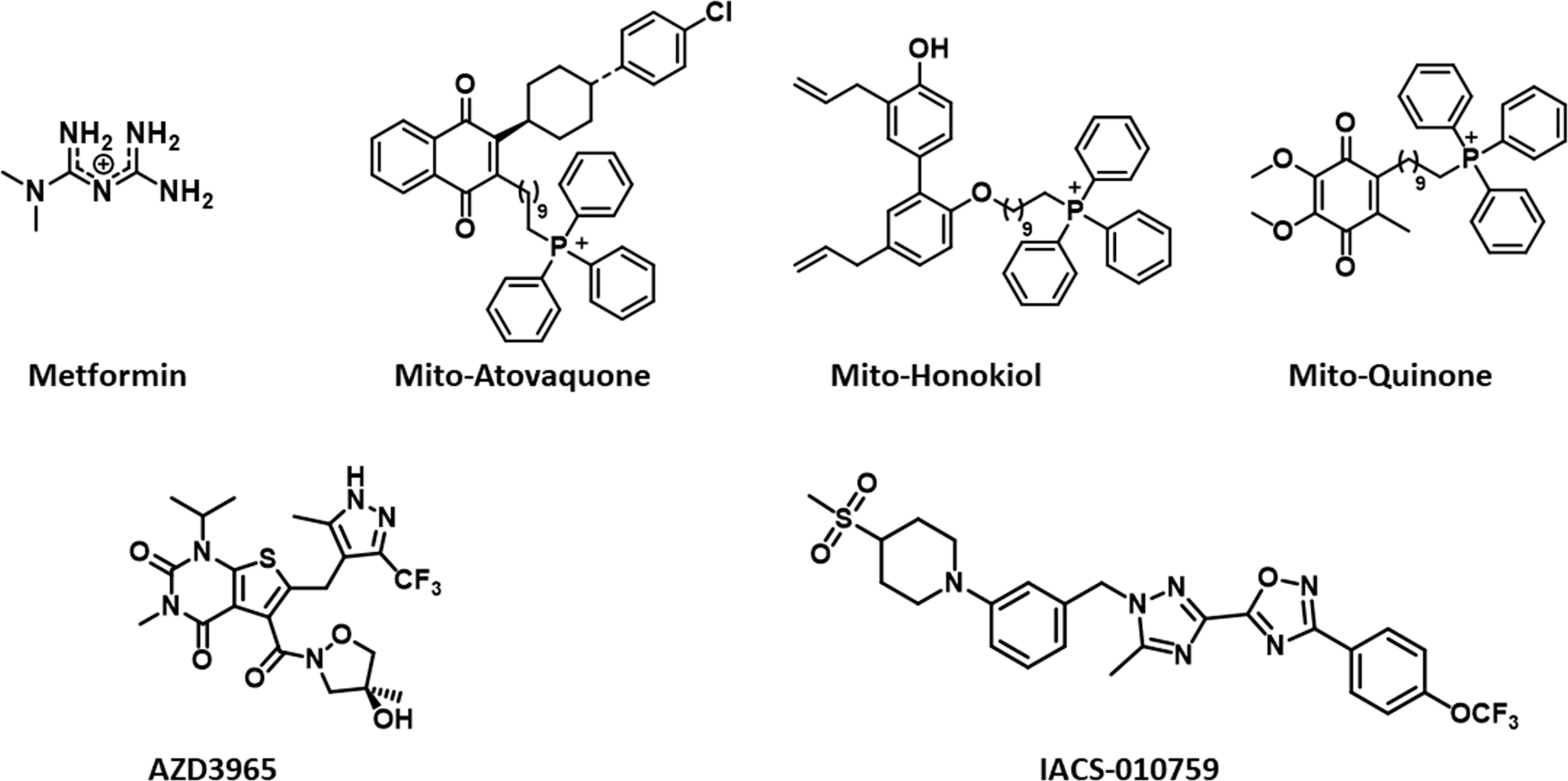
Structures of mitochondria-targeted natural products, a co-enzyme, and drugs.

**Figure 3:**
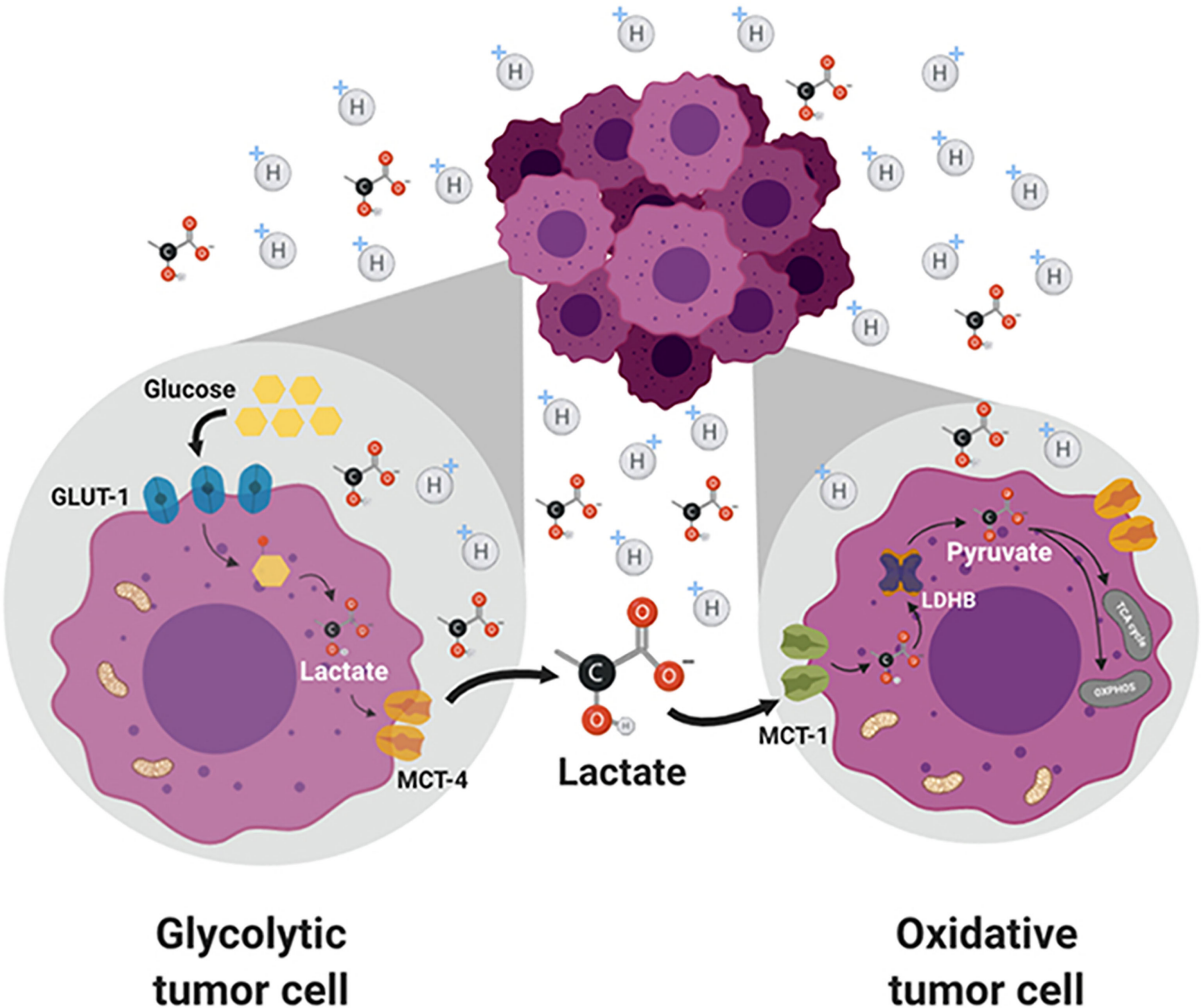
Lactate exchange between the glycolytic tumor cells present in the hydrophobic core and the oxidative tumor cells. Lactate transported out of the glycolytic cells is taken up by the oxidative cells and used for energy production. Figure licensed under CC BY, copyright ^©^ 2019 de la Cruz-López, Castro-Muñoz, Reyes-Hernández, García-Carrancá and Manzo-Merino. Lactate in the Regulation of Tumor Microenvironment and Therapeutic Approaches. Front Oncol. 2019 Nov 1;9:1143.

Circulating lactate is a prominent source of energy in highly metastatic melanoma cells. These cells have higher levels of MCT1 (25). MCT1 inhibition by AZD3965 (**Figure 2**) inhibits metastasis in part by increasing oxidative stress, depleting glutathione, and producing ROS (26). Antioxidants or drugs enhancing intracellular glutathione (*e.g.*, N-acetylcysteine supplementation) negated the effect of AZD3965. Supplementation with vitamin E, a lipid peroxidation chain-breaking antioxidant, enhanced tumor progression in xenografts. Metastatic melanoma cells undergo increased oxidative stress, and antioxidants protect them against oxidative stress (25). MCT1 expression was shown to be correlated with upregulation of glycolysis in tumor cells, and MCT1 inhibition was shown to impair glycolysis and upregulate mitochondrial metabolism (9, 27). NMR spectroscopy using ^13^C-labeled substrates showed reactivation of pyruvate metabolism during MCT1 inhibition (28). In the *in vivo* setting in mice xenografts, AZD3965 improved tumor bioenergetics that was monitored noninvasively using *in vivo* ^31^P NMR. Most tumors are heterogeneous, composed of glycolytic cells, oxidative cells, and stromal cells. Glycolytic tumor cells are located at the hydrophobic core and use glucose to form lactate. Oxidative tumor cells are located closer to the vasculature; they use lactate as an oxidative fuel and oxidative phosphorylation (OXPHOS) to generate ATP (21). This makes more glucose available for glycolytic tumor cells. Lactate exchange occurs between tumor cells. Hypoxic cancer cells provide lactate as an energy source for mitochondrial respiration in oxidative cells in a process termed as “metabolic symbiosis” (**Figure 3**) (26). Targeting lactate-driven respiration selectively killed hypoxic tumor cells in mice (29). Of the several monologs of MCTs, MCT1 and MCT4 are the most studied in human cancers. Unlike MCT1, MCT4 is upregulated by hypoxia through a hypoxia-inducible factor 1-alpha–dependent mechanism (30). Myc oncoproteins regulate MCT1 levels (31). Metabolic shift to OXPHOS is a mechanism of resistance to MCT1 inhibitors, and metformin overcomes this resistance. These studies support the use of MCT1 inhibitors in combination with OXPHOS inhibitors in therapies for MCT1 and Myc-overexpressing malignancies (9, 32). MCT1 inhibitors decrease nicotinamide adenine dinucleotide phosphate and glutathione levels, and enhance intracellular ROS. Prooxidant therapies were suggested to augment the potency of lactate transport inhibitors. Dual inhibition of the lactate transport and OXPHOS mechanism were shown to exert synthetic lethality in cancer cells (33).

Pyruvate is reduced to lactate, and the NADH (nicotinamide adenine dinucleotide [NAD] plus hydrogen) cofactor is oxidized to NAD^+^ by lactate dehydrogenase A. This is a thermodynamically favored reaction. Lactate can be oxidized to pyruvate using NAD^+^ in a reaction catalyzed by the enzyme, lactate dehydrogenase B. Lactate has emerged as more than just a by-product or the waste product of the glycolytic metabolism in cancer cells (21). It is responsible for the highly acidic TIME that triggers many of the hallmarks of cancer, and is proposed as an important oncometabolite (26). Depriving tumor cells of their ability to export lactate is a potentially promising therapeutic strategy. Glycolytic tumors express MCT4 to excrete lactate to prevent intracellular over acidification. Oxidative tumors overexpress MCT1 to acquire more lactate for energy. An MCT inhibitor, AZD3965, is being evaluated in clinical trials (34). The selective inhibition of lactate transport by AZD3965 presents a novel way of targeting the metabolic phenotype in tumors that preferentially express MCT1. AZD3965 increased intratumor lactate concentration and the pH. Excess production of lactate is removed from cells *via* MCT. The directionality of transport by MCTs depends on lactate and proton concentration gradients (35). Lactate is involved in tumor invasion and immune suppression (36). AZD3965 was shown to increase oxidative metabolism in tumor cells (28). Metformin, a weak OXPHOS inhibitor, enhanced the antiproliferative potency of AZD3965 (28).

Metformin, a drug used to treat diabetes, has decreased the incidence of cancers in patients taking the treatment (37). Metformin has been shown to improve the survival rates of breast cancer patients (38). Although its precise mechanism of action is still unclear, metformin is thought to act by inhibiting the NADH dehydrogenase enzyme associated with complex I (39).

Mitochondria play a critical role in cancer biology and cancer therapy through macromolecule synthesis and energy production (40). Mitochondrial respiration supports ATP production and is also essential for tumorigenesis and tumor progression. Malignant tumors selectively retain the mitochondrial genome and electron transport chain (41). Tumors with mitochondrial DNA mutation are benign, indicating the importance of respiration to tumor progression. Targeting mitochondrial metabolism presents a relatively new concept in effective cancer therapeutics (42). In addition to exerting bioenergetic functions, mitochondria provide the building blocks for tumor metabolism, redox control, and calcium; they also regulate metabolism, apoptosis, and cell survival.

## OXPHOS as a Potential Target in Cancer Therapy

The mitochondrial membrane consists of a porous outer membrane coupled with a protein-rich inner membrane. To cross the mitochondrial membrane, molecules are required to overcome the activation energy associated with water molecules. In molecules conjugated to the triphenylphosphonium cation (TPP^+^), the positive charge is delocalized over the three phenyl rings. This lowers the activation energy barrier, allowing these molecules to penetrate the membrane and enter the mitochondria efficiently (43–45). The hyperpolarized mitochondrial membrane of a cancer cell (−220 mV) compared with that of a healthy mitochondrion (−160 mV) facilitates the rapid and selective entry of molecules with a positive charge into mitochondrion (46).

**Figure 4:**
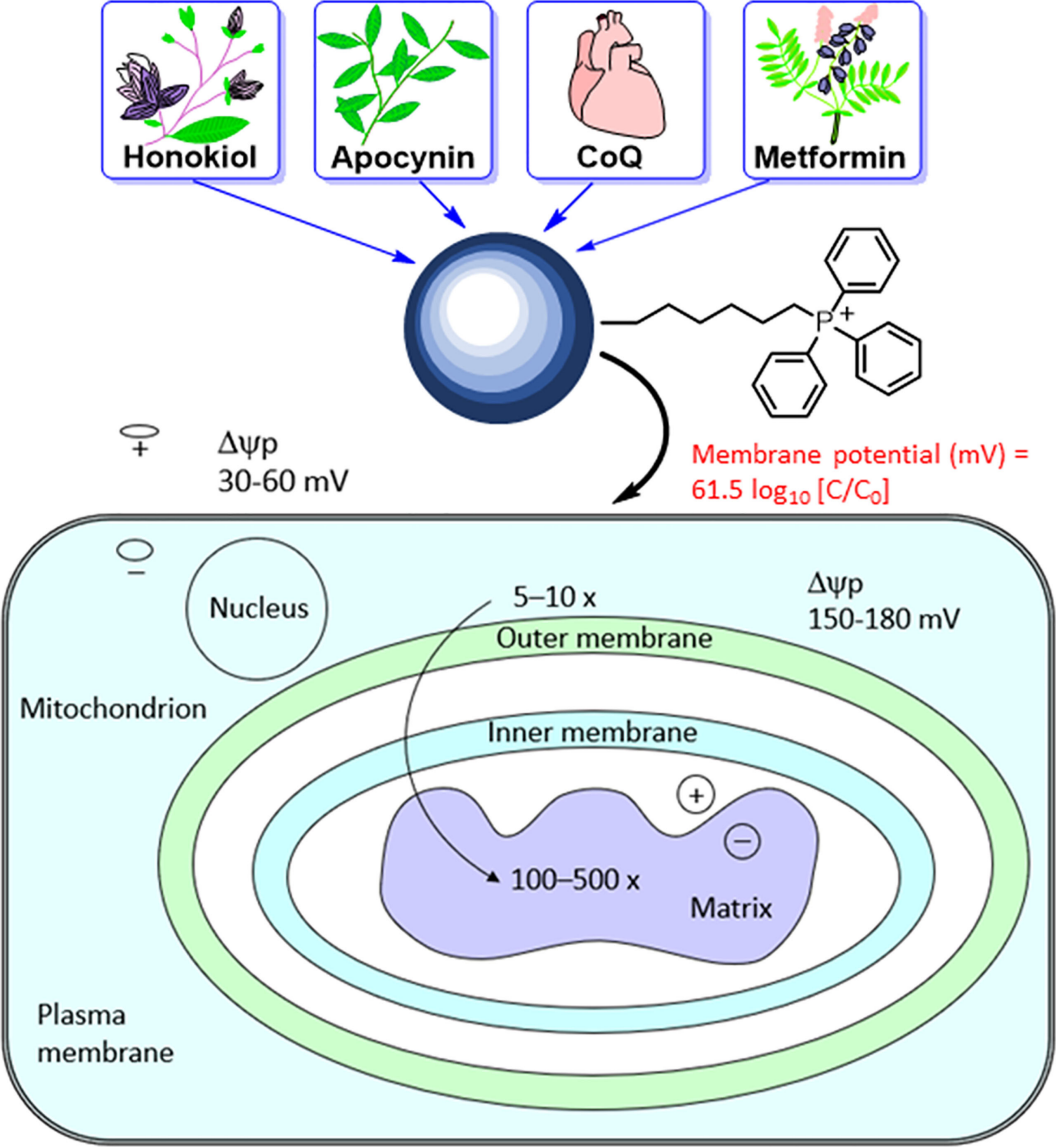
Mitochondrial uptake of TPP^+^-containing drugs and natural products into cancer cells due to enhanced hyperpolarization. The top portion of this figure is reprinted (adapted) with permission from Zielonka J, Joseph J, Sikora A, Hardy M, Ouari O, Vasquez-Vivar J, Cheng G, Lopez M, Kalyanaraman B Mitochondria-Targeted Triphenylphosphonium-Based Compounds: Syntheses, Mechanisms of Action, and Therapeutic and Diagnostic Applications. Chem Rev. 2017 Aug 9;117(15):10043-10120. Copyright 2017 American Chemical Society. The bottom portion of this figure is adapted from Smith RA, Porteous CM, Gane AM, Murphy MP. Delivery of bioactive molecules to mitochondria *in vivo*. Proc Natl Acad Sci U S A 2003 Apr 29;100(9):5407-12. Copyright 2003 National Academy of Sciences.

The most studied and widely used mitochondria-targeting vector is TPP^+^ (47–50). TPP^+^ possesses a single positive charge that is delocalized over three phenyl groups, stabilizing resonance. In addition to the charge, the hydrophobicity of the lipophilic cation favors the interaction with the hydrophobic inner mitochondrial membrane. Driven by the membrane potential, the concentration of the TPP^+^ in the cytoplasm increases by about 5–10 fold, compared with that of the extracellular space. The resulting accumulation of TPP^+^ in the cytoplasm is about 100–500 times that in the extracellular space (**Figure 4**). This provides a highly targeted and effective mitochondrial vector. The advantages of TPP^+^-based targeting of molecules are the stability of TPP^+^ in the biological system, low chemical reactivity toward cellular components, combination of lipophilic and hydrophilic moieties, and ease of synthesizing large quantities of molecules for *in vivo* work (43).

Reports indicate that a decrease in the core body temperature and death result from the excessive inhibition of OXPHOS (51). Mitochondria-targeted honokiol did not elicit these effects (52). Specific inhibitors of complex I of the mitochondrial electron transport chain in cancer cells are emerging as anticancer agents in hypoxic tumors (7, 51). Hypoxic tumors with a reduced capacity for compensatory glycolysis may be more susceptible to OXPHOS inhibitors. Do all complex I inhibitors have the potential to become anticancer agents? The lack of effect of complex I inhibitors on normal cells must be studied in all cases (rotenone has off-target pharmacology). It is likely that the mechanism of the inhibitory activity of IACS-010759 (**Figure 2**) in complex I differs from that of the other known quinone site inhibitors (53).

Previously, using low-temperature electron paramagnetic resonance, we determined the mitochondrial redox changes in pancreatic cancer cells treated with TPP^+^-containing mitochondria-targeted agents (54). Based on the electron paramagnetic resonance spectral changes of complex I iron-sulfur (FeS) clusters, [2Fe2S]^+^ and [4Fe-4S]^+^, we surmised that TPP^+^-containing mitochondria-targeted drugs (complex I inhibitors) bind closer to the NADH-dehydrogenase site in the mitochondrial complex I dictated by the NAD^+^/NADH couple (55). In contrast with other mitochondria-targeted drugs (*e.g.*, Mito-honokiol, Mito-Q), Mito_10_-atovaquone (Mito_10_-ATO) inhibits both complex III- and complex I-induced oxygen consumption (56). Recent reports indicate that selective targeting and inhibiting of mitochondrial complex III mitigate and reverse immunosuppression by T_regs_, promoting the function of effector T cells (57). T_regs_ suppress antitumor immunity that greatly hampers immunotherapy. Inhibitors of mitochondrial complex III (antimycin A) and not complex I (rotenone) reversed the immunosuppressive function of T_regs_ (57). Although several relatively nontoxic mitochondrial complex I inhibitors are available (excluding rotenone that is toxic), the availability of complex III inhibitors is relatively scarce except for antimycin A and atovaquone. We showed, for the first time, that conjugating atovaquone to TPP^+^ and increasing the aliphatic linker side chain length generates Mito-ATO analogs (*e.g.*, Mito_4_-ATO and Mito_10_-ATO) that are potent inhibitors of complex I- and complex III-induced oxygen consumption in cancer cells (56). Mito_4_-ATO and Mito_10_-ATO effectively inhibit T_reg_ differentiation and survival while stimulating effector T cell response. These compounds represent a new class of antitumor and immunoregulatory drugs targeting OXPHOS, one of the key vulnerabilities of cancer cells.

### Therapeutic Targeting of Tumor Hypoxia

**Figure 5:**
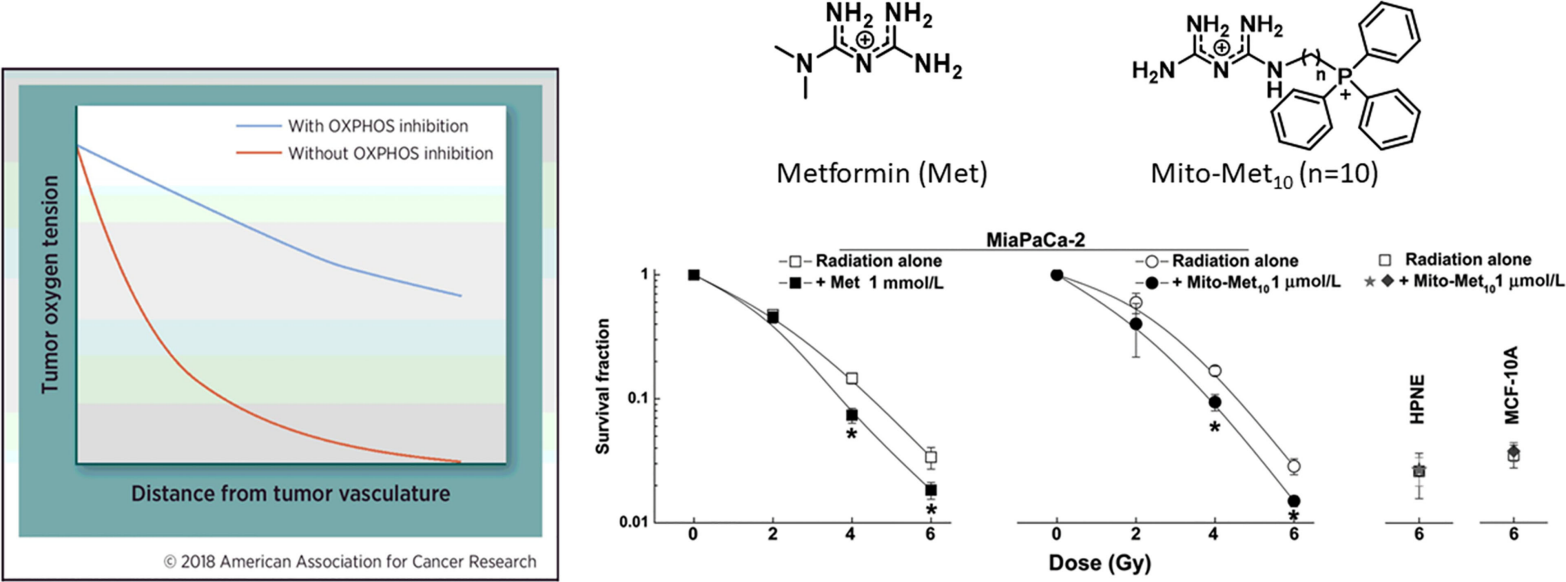
Mitochondria-targeted metformin enhances oxygen concentration in pancreatic cancer cells by inhibiting mitochondrial respiration and makes these cells more vulnerable to radiation. *, *P* < 0.05 compared with radiation alone. The left portion of the figure is reprinted from Clinical Cancer Research, 2018, 24/11, 2482-2490, Ashton TM, McKenna WG, Kunz-Schughart LA, Higgins GS, Oxidative Phosphorylation as an Emerging Target in Cancer Therapy, with permission from AACR. The right portion of the figure is adapted from Cancer Research, 2016, 76/13, 3904-3915, Cheng G, Zielonka J, Ouari O, Lopez M, McAllister D, Boyle K, Barrios CS, Weber JJ, Johnson BD, Hardy M, Dwinell MB, Kalyanaraman B, Mitochondria-Targeted Analogues of Metformin Exhibit Enhanced Antiproliferative and Radiosensitizing Effects in Pancreatic Cancer Cells, with permission from AACR.

An effective approach to decrease tumor hypoxia (increase oxygen levels in tumor tissues) is to inhibit mitochondrial respiration and decrease oxygen consumption (**Figure 5**) (7). Thus, a vulnerable target in antitumor therapy is the tumor mitochondria. Oxygen tension plays a critical role in the response to radiation therapy (58). Higher metabolic rates in tumors result in tumor hypoxia, especially in solid tumors with disorganized vasculature (59, 60). Hypoxic tumors are not very sensitive to radiation therapy. It is estimated that hypoxic tumor cells require three times the dose of radiation needed to cause the same amount of cell death under normal oxygen tension (59, 61, 62). Hypoxic tumors can, however, be sensitized to radiation therapy by targeting and inhibiting mitochondrial respiration (58, 63). Hypoxia is recognized as a hallmark of the TIME. As we have shown with Mito-metformin (64), inhibition of mitochondrial respiration enhances radiation-induced pancreatic cancer cell killing, attributable to increased oxygen tension or decreased hypoxia. Several FDA-approved drugs (papaverine, atovaquone, metformin) that inhibit mitochondrial respiration are now undergoing clinical trials as radiation sensitizers in radiation therapy of tumors (58, 64). Atovaquone increased tumor oxygenation and inhibited hypoxic gene expression in lung cancer patients (65). In a recent clinical trial, a combination of blood-oxygen-level-dependent functional MRI and circulating markers of hypoxia are used to demonstrate whether atovaquone results in decreased tumor hypoxia (NCT02628080) (66). If this approach is successful, other more-potent mitochondria-targeted drugs, such as Mito-honokiol, Mito-magnolol, Mito-ATO, and Mito-hydroxyurea, may be used as potential radiation sensitizers.

### Suppression of Drug-Resistant Tumors by OXPHOS Inhibition

**Figure 6:**
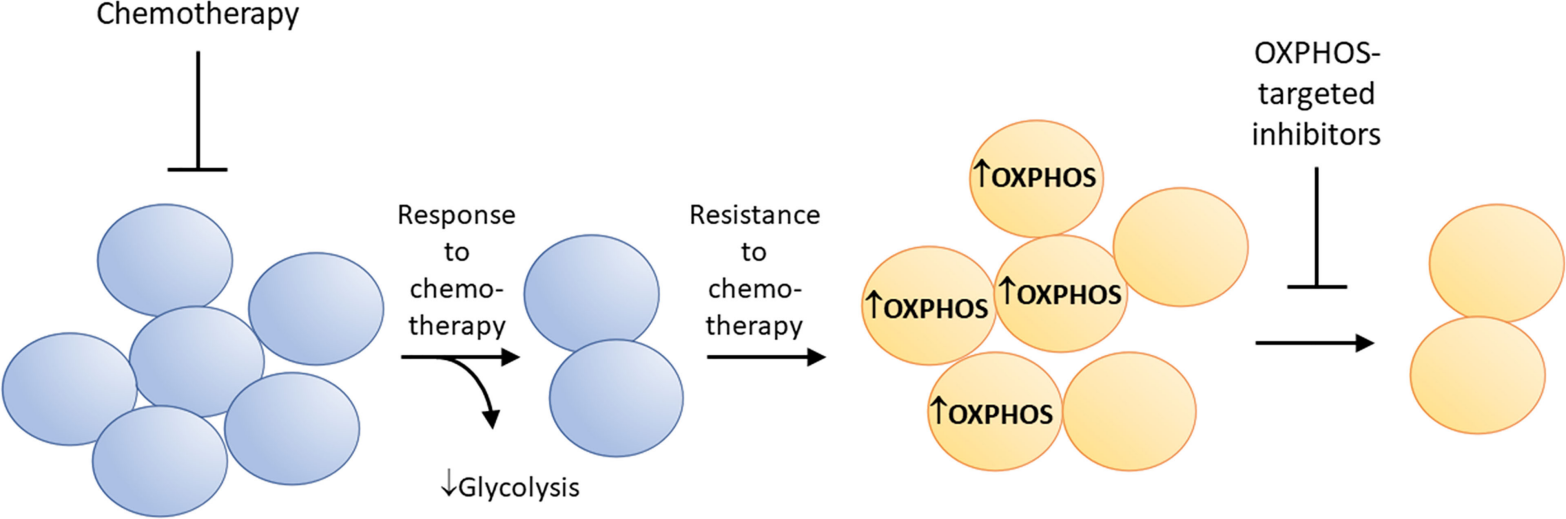
Metabolic reprogramming from glycolysis to enhanced OXPHOS occurs in drug-resistant cancer cells. This characteristic makes these cells more vulnerable to mitochondria-targeted OXPHOS inhibitors.

Metabolic reprogramming has been linked to therapeutic resistance in several cancer cell types (67). Some drug-resistant cancer cells are highly sensitive to OXPHOS inhibition (7, 68–70). This is partly attributed to metabolic reprogramming from glycolysis to enhanced mitochondrial OXPHOS. Tyrosine kinase inhibition results in drug resistance and metabolic reprogramming in tumor cells (71). **Figure 6** shows how kinase inhibitors induce metabolic reprogramming (enhanced OXPHOS) and therapeutic resistance in cancer cells, and how concomitant OXPHOS inhibition can overcome resistance to kinase inhibition. Ibrutinib-resistant myeloma cancer cells rely on OXPHOS and glutaminolysis for energy. Small molecule inhibitors of OXPHOS (IACS-010759) inhibit proliferation of Ibrutinib-resistant cells. The Phase 1 trial investigates the side effects and best dose of IACS-010759 in treating relapsed or refractory myeloma patients (NCT03291938) (72).

## Targeting Vulnerabilities in the TIME

Tumor cells create a nutrient-depleted, oxygen-deficient, and acidic microenvironment. This environment makes it difficult for T cells to survive and attack the tumor cells (73). Factors such as tumor hypoxia, acidity, and lactate transport that are vulnerable to tumor cells also pose vulnerability in the TIME. Immune checkpoint blockade therapy, while very effective in some cancer patients, fails in most cancer patients (74). The major of factors that limit the success of this therapy are the heterogeneity of the components that make up the TIME (75).

The TIME is considered as a vulnerable therapeutic target in cancer treatment. Rationalized targeting of the TIME was proposed in 2020 (76). An acidic milieu is one of the hallmarks of cancer. Approaches to reverse the acidification of the TIME could facilitate chemotherapy and immunotherapy (L-DOS47) (77, 78). L-DOS47 is an antineoplastic immunoconjugate consisting of a recombinant camelid single-domain antibody that recognizes an antigen-related cell adhesion molecule and the enzyme urease derived from the *Canavalia ensiformis* (or jack bean) plant. This adhesion molecule (CEACAM6) is highly expressed in certain tumor cells. Upon intravenous administration, the antibody fragment moiety of L-DOS47 binds to CEACAM6, and the urease part of L-DOS47 catalyzes the hydrolysis of urea into ammonia. High levels of ammonia alkalinize the acidic TIME, increasing the pH of the TIME.

Emerging research shows the important role of lactate as a “signaling molecule” in regulating cancer cell survival, proliferation, and metastasis (79, 80). Current studies also indicate that lactate is emerging as a key immunosuppressive metabolite promoting the escape of immune surveillance in hypoxic tumors (73). Lactate generated by hypoxic tumor cells was found to strongly inhibit the antitumor immune response through attenuation of the cytotoxic activity of human cytotoxic T lymphocyte and natural killer cells. Lactate also stimulates myeloid-derived suppressor cells in tumors, which inhibit natural killer cell cytotoxicity. More recent findings reveal that preventing T_regs_ from feeding on lactate slows tumor growth and increases response to immunotherapy (24, 81). The drug of choice is AZD3965, which inhibits the MCT1 transporter protein. Although lactate was once considered to be a waste product, it is now thought to contribute to the onset and progression of cancer, favoring metastasis and tumor angiogenesis. In the TIME, lactate induces the metabolic coupling between cancer cells, immune cells, and stroma cells. Lactate participates in immune evasion through inhibition of T cells and induction of the M2 macrophage polarization attributed to tumor progression (82, 83).

## Activators of AMPK and Regulation of Immunosuppressive Activity

AMP-activated protein kinase (AMPK), a master regulator of cellular energy homeostasis, is typically activated by increased intracellular AMP (84, 85). We previously showed that OXPHOS inhibitors stimulate a signaling pathway for antiproliferative effects, linking complex I inhibition to AMPK activation (52, 53, 64, 86, 87) and leading to the inhibition of signal transducer and activator of transcription 3 (STAT3) ser727 phosphorylation (88). AMPK activation inhibits the functions of myeloid-derived suppressor cells (89–92). The mitochondria-targeted drug, phenformin, inhibits myeloid-derived suppressor cells and enhances the antitumor activity (93). Cumulative evidence suggests that STAT3 activation leads to immunosuppression, and that inhibiting STAT3 signaling is an effective strategy to improve antitumor immunity (94, 95).

## Targeting OXPHOS in Tumors Remodels the TIME

Phosphoinositide 3-kinase (P13K) inhibitors increased mitochondrial trafficking and enhance tumor invasion (96). This was a paradoxical and unexpected effect of PI3K inhibitors. The resistance to PI3K inhibitors in cancer patients was attributed to the spatiotemporal induction in mitochondrial respiration, enhancing ATP production and fueling the increased energetic needs for metastasis. However, mitochondria-targeted drugs could thwart mitogenesis (96). Gamitrinib, a small-molecule, mitochondria-targeted Hsp90 (heat shock protein 90) inhibitor, and other OXPHOS inhibitors abrogated the increase in tumor cell invasion induced by PI3K inhibitors. This study revealed that the metabolic reprogramming (enhanced OXPHOS) that occurs in metastatic cancer cells is responsible for metastatic cancer cell survival and progression. OXPHOS is potentially a druggable antimetastatic target. Reports indicate that an OXPHOS inhibitor, IACS-010759, inhibits melanoma brain metastasis (97). The complex I inhibitor also inhibits myeloid-derived suppressor cells in the metastatic TIME. TPP^+^-conjugated OXPHOS inhibitors of complex I and complex III—Mito-ATO, Mito-magnolol, and Mito-hydroxyurea—are potentially suitable anti-metastatic drugs (56, 87). It was reported that brain metastases from patients with melanoma displayed a considerable degree of immunosuppression and increased expression of genes related to OXPHOS. IACS-010759, a reported complex I inhibitor, blocked metastases formation in mouse models (97). Mitochondria-targeted tamoxifen is currently undergoing clinical trial for treating conventional therapy resistant breast cancer (98).

Mitochondrial ROS are proposed as redox signaling species (99). Mitochondria-targeted agents inhibit tumorigenesis in colon cancer cells through inhibition of mitochondrial redox signaling (18). TPP^+^-containing mitochondria-targeting agents (*e.g.*, Mito-metformin) inhibit pancreatic cancer cell proliferation *via* downregulation of the Akt/FOXO3/FOXM1 signaling axis in pancreatic cancer cells. Mito-lonidamine inhibits complex I-mediated oxygen consumption, oxidation of peroxiredoxin Akt/mTOR/p7056K signaling, and induction of autophagy in lung cancer cells; Mito-magnolol decreases complex I-mediated oxygen consumption and Akt/FOXO signaling, and blocks cell cycle progression and cell proliferation in melanoma cells; and Mito-honokiol inhibits lung cancer cell proliferation through activation of AMPK and inhibition of STAT3 signaling (52). Pyrvinium pamoate and other FDA-approved drugs (*e.g.*, trifluoperazine, mitoxantrone) inhibit cancer stem cell respiration (88).

Mitochondrial dynamics (fusion and fission) are involved in controlling mitochondrial respiration. Pancreatic ductal adenocarcinoma cells are highly fragmented and essential to oncogenicity (100). This mitochondrial feature was recognized as a metabolic vulnerability in tumors. Normalizing the fragmented mitochondria *via* mitochondrial fusion decreases OXPHOS, which correlates with suppressed tumor growth (100). Inhibition of dynamin-related protein 1 or overexpression of mitofusin 2 using an FDA-approved arthritis drug (*e.g.*, leflunomide) decreased OXPHOS and suppressed tumor growth. Fusion is a specific and druggable target in mitochondria.

## The Role of the TIME in Cancer Health Disparities: Effect of Mitochondria-Targeted Drugs

There is increasing evidence for the existence of racial and ethnic disparities in the breast cancer TIME (101). Higher levels of pro-tumorigenic factors (*e.g.*, macrophages, T_regs_, exhausted T cells) were identified in the TIME of Black breast cancer patients as compared with white counterparts (101–103). Upregulation of genes associated with OXPHOS was identified in tumor samples obtained from Black cancer patients (104). Tumors from Black patients have more mitochondria, estrogen-related receptor 1, and peroxisome proliferator-activated receptor-gamma coactivator-1alpha (105). Clinical trial data show that Black cancer patients respond better to mitochondrial inhibitors (*e.g.*, metformin) than white cancer patients (106). Developing the next generation of mitochondrial inhibitors was perceived to be a promising therapeutic strategy to mitigate or prevent enhanced mortality in Black cancer patients (104). It is conceivable that newly developed, mitochondria-targeted modified natural products, a co-enzyme, and FDA-approved drugs (*e.g.*, Mito-honokiol, Mito-magnolol, Mito-Q, Mito-ATO, Mito-metformin, and Mito-hydroxyurea) and their analogs potentially could be useful in addressing the cancer health disparities.

## Summary and Future Perspectives

Mitochondria are a vulnerable target for cancer therapy. Inventing new small molecules and therapeutic approaches selectively targeted to tumor mitochondria is an emerging area of research. Cancer cells use both glycolysis and mitochondrial respiration for growth, progression, and metastasis. This is achieved through metabolic reprogramming between glycolysis and mitochondrial oxidative metabolism. Metabolic reprogramming is a dynamic and metabolically flexible process that changes during the different stages of cancer (tumor initiation, growth, progression, metastasis, and resistance to therapy).

Mitochondria-targeted drugs based on TPP^+^ and natural products are potent, tumor selective, and relatively nontoxic (with minimal off-target pharmacology) in cells and preclinical tumor xenografts. Several natural products conjugated to the TPP^+^ moiety (*e.g.*, Mito-honokiol, Mito-magnolol) exhibit significantly more antiproliferative potency than other non-TPP^+^ mitochondrial inhibitors (*e.g.*, metformin, phenformin, IACS-010759) in multiple cancer cells. Mitochondria-targeted drugs combined with standard-of-care chemotherapy, radiation therapy, and immunotherapy are likely to have potentiating effects in cancer treatment. These combinational modalities may counteract the immunosuppressive microenvironment and enhance immunotherapy using the checkpoint inhibitors. The TIME in Black cancer patients consists of more pro-tumorigenic factors and mitochondrial dysfunction than in white cancer patients. Specific alterations in OXPHOS gene expression in tumors may be used as a biomarker for enhanced sensitivity of mitochondria-targeted drugs. Thus, developing a highly potent, less toxic, and tumor/TIME-selective next generation of mitochondrial OXPHOS inhibitors is timely and critical in exploiting tumor/TIME vulnerability and addressing the novel role of mitochondrial biology in cancer health disparities.

## Author Contributions

BK drafted the manuscript. GC performed the mitochondrial bioenergetics experiments. MH synthesized and purified all of the mitochondria-targeted inhibitors. BK, GC, and MH reviewed and revised the manuscript. All authors contributed to the article and approved the submitted version.

## Funding

The research reported in this publication was supported by the National Cancer Institute of the National Institutes of Health under Award Numbers R01CA208648 and R01CA232433. The content is solely the responsibility of the authors and does not necessarily represent the official views of the National Institutes of Health.

## Conflict of Interest

BK and MH are co-inventors of US Patent No. 10,836,782/European Patent No. 3307254, “Mito-honokiol compounds and methods of synthesis and use thereof,” and BK is an inventor of US Patent No. US8962600B2, “Neuroprotective compounds and their use,” for use of Mito-honokiol and Mito-apocynin, respectively, in cancer and Parkinson’s disease.

## Publisher’s Note

All claims expressed in this article are solely those of the authors and do not necessarily represent those of their affiliated organizations, or those of the publisher, the editors and the reviewers. Any product that may be evaluated in this article, or claim that may be made by its manufacturer, is not guaranteed or endorsed by the publisher.

## References

[1] Boehm JS Garnett MJ Adams DJ Francies HE Golub TR Hahn WC. Cancer Research Needs a Better Map. Nature (2021) 589(7843):514–6. doi: 10.1038/d41586-021-00182-0 33500573

[2] Hirschhaeuser F Sattler UG Mueller-Klieser W. Lactate: A Metabolic Key Player in Cancer. Cancer Res (2011) 71(22):6921–5. doi: 10.1158/0008-5472.Can-11-1457 22084445

[3] Brand MD. Riding the Tiger - Physiological and Pathological Effects of Superoxide and Hydrogen Peroxide Generated in the Mitochondrial Matrix. Crit Rev Biochem Mol Biol (2020) 55(6):592–661. doi: 10.1080/10409238.2020.1828258 33148057

[4] Grasso D Zampieri LX Capelôa T Van de Velde JA Sonveaux P. Mitochondria in Cancer. Cell Stress (2020) 4(6):114–46. doi: 10.15698/cst2020.06.221 PMC7278520 32548570

[5] Lau AN Vander Heiden MG. Metabolism in the Tumor Microenvironment. Annu Rev Cancer Biol (2020) 4(1):17–40. doi: 10.1146/annurev-cancerbio-030419-033333

[6] Crunkhorn S. Targeting the Mitochondria to Block Tumour Growth. Nat Rev Drug Discovery (2021) 20(2):97. doi: 10.1038/d41573-021-00001-1 33398166

[7] Ashton TM McKenna WG Kunz-Schughart LA Higgins GS. Oxidative Phosphorylation as an Emerging Target in Cancer Therapy. Clin Cancer Res (2018) 24(11):2482–90. doi: 10.1158/1078-0432.Ccr-17-3070 29420223

[8] Vasan K Werner M Chandel NS. Mitochondrial Metabolism as a Target for Cancer Therapy. Cell Metab (2020) 32(3):341–52. doi: 10.1016/j.cmet.2020.06.019 PMC7483781 32668195

[9] Doherty JR Yang C Scott KE Cameron MD Fallahi M Li W. Blocking Lactate Export by Inhibiting the Myc Target MCT1 Disables Glycolysis and Glutathione Synthesis. Cancer Res (2014) 74(3):908–20. doi: 10.1158/0008-5472.Can-13-2034 PMC3946415 24285728

[10] Zhu Y Dean AE Horikoshi N Heer C Spitz DR Gius D. Emerging Evidence for Targeting Mitochondrial Metabolic Dysfunction in Cancer Therapy. J Clin Invest (2018) 128(9):3682–91. doi: 10.1172/jci120844 PMC6118595 30168803

[11] Altman BJ Stine ZE Dang CV. From Krebs to Clinic: Glutamine Metabolism to Cancer Therapy. Nat Rev Cancer (2016) 16(10):619–34. doi: 10.1038/nrc.2016.71 PMC5484415 27492215

[12] Wang H Liu C Zhao Y Gao G. Mitochondria Regulation in Ferroptosis. Eur J Cell Biol (2020) 99(1):151058. doi: 10.1016/j.ejcb.2019.151058 31810634

[13] Liu Y.E. Shi Y. Mitochondria as a Target in Cancer Treatment. MedComm (2020) 1(2):129–39. doi: 10.1002/mco2.16 PMC8491233 34766113

[14] Vander Heiden MG Cantley LC Thompson CB. Understanding the Warburg Effect: The Metabolic Requirements of Cell Proliferation. Science (2009) 324(5930):1029–33. doi: 10.1126/science.1160809 PMC2849637 19460998

[15] Kalyanaraman B. Teaching the Basics of Cancer Metabolism: Developing Antitumor Strategies by Exploiting the Differences Between Normal and Cancer Cell Metabolism. Redox Biol (2017) 12:833–42. doi: 10.1016/j.redox.2017.04.018 PMC5406543 28448945

[16] Warburg O Wind F Negelein E. THE METABOLISM OF TUMORS IN THE BODY. J Gen Physiol (1927) 8(6):519–30. doi: 10.1085/jgp.8.6.519 PMC2140820 19872213

[17] Koppenol WH Bounds PL Dang CV. Otto Warburg's Contributions to Current Concepts of Cancer Metabolism. Nat Rev Cancer (2011) 11(5):325–37. doi: 10.1038/nrc3038 21508971

[18] Weinberg F Hamanaka R Wheaton WW Weinberg S Joseph J Lopez M. Mitochondrial Metabolism and ROS Generation are Essential for Kras-Mediated Tumorigenicity. Proc Natl Acad Sci United. States America (2010) 107(19):8788–93. doi: 10.1073/pnas.1003428107 PMC2889315 20421486

[19] Shiratori R Furuichi K Yamaguchi M Miyazaki N Aoki H Chibana H. Glycolytic Suppression Dramatically Changes the Intracellular Metabolic Profile of Multiple Cancer Cell Lines in a Mitochondrial Metabolism-Dependent Manner. Sci Rep (2019) 9(1):18699. doi: 10.1038/s41598-019-55296-3 31822748 PMC6904735

[20] Oshima N Ishida R Kishimoto S Beebe K Brender JR Yamamoto K. Dynamic Imaging of LDH Inhibition in Tumors Reveals Rapid *In Vivo* Metabolic Rewiring and Vulnerability to Combination Therapy. Cell Rep (2020) 30(6):1798–1810.e1794. doi: 10.1016/j.celrep.2020.01.039 32049011 PMC7039685

[21] Chen YJ Mahieu NG Huang X Singh M Crawford PA Johnson SL. Lactate Metabolism is Associated With Mammalian Mitochondria. Nat Chem Biol (2016) 12(11):937–43. doi: 10.1038/nchembio.2172 PMC5069139 27618187

[22] Faubert B Li KY Cai L Hensley CT Kim J Zacharias LG. Lactate Metabolism in Human Lung Tumors. Cell (2017) 171(2):358–371.e359. doi: 10.1016/j.cell.2017.09.019 28985563 PMC5684706

[23] Hensley CT Faubert B Yuan Q Lev-Cohain N Jin E Kim J. Metabolic Heterogeneity in Human Lung Tumors. Cell (2016) 164(4):681–94. doi: 10.1016/j.cell.2015.12.034 PMC4752889 26853473

[24] Multhoff G Vaupel P. Lactate-Avid Regulatory T Cells: Metabolic Plasticity Controls Immunosuppression in Tumour Microenvironment. Signal Transduct. Target. Ther (2021) 6(1):171. doi: 10.1038/s41392-021-00598-0 33931598 PMC8087677

[25] Tasdogan A Faubert B Ramesh V Ubellacker JM Shen B Solmonson A. Metabolic Heterogeneity Confers Differences in Melanoma Metastatic Potential. Nature (2020) 577(7788):115–20. doi: 10.1038/s41586-019-1847-2 PMC6930341 31853067

[26] de la Cruz-López KG Castro-Muñoz LJ Reyes-Hernández DO García-Carrancá A Manzo-Merino J. Lactate in the Regulation of Tumor Microenvironment and Therapeutic Approaches. Front Oncol (2019) 9:1143. doi: 10.3389/fonc.2019.01143 31737570 PMC6839026

[27] Birsoy K Wang T Possemato R Yilmaz OH Koch CE Chen WW. MCT1-Mediated Transport of a Toxic Molecule is an Effective Strategy for Targeting Glycolytic Tumors. Nat Genet (2013) 45(1):104–8. doi: 10.1038/ng.2471 PMC3530647 23202129

[28] Beloueche-Babari M Wantuch S Casals Galobart T Koniordou M Parkes HG Arunan V. MCT1 Inhibitor AZD3965 Increases Mitochondrial Metabolism, Facilitating Combination Therapy and Noninvasive Magnetic Resonance Spectroscopy. Cancer Res (2017) 77(21):5913–24. doi: 10.1158/0008-5472.Can-16-2686 PMC5669455 28923861

[29] Sonveaux P Végran F Schroeder T Wergin MC Verrax J Rabbani ZN. Targeting Lactate-Fueled Respiration Selectively Kills Hypoxic Tumor Cells in Mice. J Clin Invest (2008) 118(12):3930–42. doi: 10.1172/jci36843 PMC2582933 19033663

[30] Ullah MS Davies AJ Halestrap AP. The Plasma Membrane Lactate Transporter MCT4, But Not MCT1, is Up-Regulated by Hypoxia Through a HIF-1alpha-Dependent Mechanism. J Biol Chem (2006) 281(14):9030–7. doi: 10.1074/jbc.M511397200 16452478

[31] Dong Y Tu R Liu H Qing G. Regulation of Cancer Cell Metabolism: Oncogenic MYC in the Driver's Seat. Signal Transduct. Target. Ther (2020) 5(1):124. doi: 10.1038/s41392-020-00235-2 32651356 PMC7351732

[32] Stine ZE Walton ZE Altman BJ Hsieh AL Dang CV. MYC, Metabolism, and Cancer. Cancer Discovery (2015) 5(10):1024–39. doi: 10.1158/2159-8290.Cd-15-0507 PMC4592441 26382145

[33] Benjamin D Robay D Hindupur SK Pohlmann J Colombi M El-Shemerly MY. Dual Inhibition of the Lactate Transporters MCT1 and MCT4 Is Synthetic Lethal With Metformin Due to NAD+ Depletion in Cancer Cells. Cell Rep (2018) 25(11):3047–3058.e3044. doi: 10.1016/j.celrep.2018.11.043 30540938 PMC6302548

[34] Cancer Research UK. A Phase I Trial of AZD3965 in Patients With Advanced Cancer [Online] (2021). Available at: https://clinicaltrials.gov/ct2/show/NCT01791595.

[35] Payen VL Hsu MY Rädecke KS Wyart E Vazeille T Bouzin C. Monocarboxylate Transporter MCT1 Promotes Tumor Metastasis Independently of Its Activity as a Lactate Transporter. Cancer Res (2017) 77(20):5591–601. doi: 10.1158/0008-5472.Can-17-0764 28827372

[36] Romero-Garcia S Moreno-Altamirano MM Prado-Garcia H Sánchez-García FJ. Lactate Contribution to the Tumor Microenvironment: Mechanisms, Effects on Immune Cells and Therapeutic Relevance. Front Immunol (2016) 7:52. doi: 10.3389/fimmu.2016.00052 26909082 PMC4754406

[37] Morales DR Morris AD. Metformin in Cancer Treatment and Prevention. Annu Rev Med (2015) 66:17–29. doi: 10.1146/annurev-med-062613-093128 25386929

[38] Vancura A Bu P Bhagwat M Zeng J Vancurova I. Metformin as an Anticancer Agent. Trends Pharmacol Sci (2018) 39(10):867–78. doi: 10.1016/j.tips.2018.07.006 PMC6153060 30150001

[39] Vial G Detaille D Guigas B. Role of Mitochondria in the Mechanism(s) of Action of Metformin. Front Endocrinol (Lausanne) (2019) 10:294. doi: 10.3389/fendo.2019.00294 31133988 PMC6514102

[40] Murphy MP Hartley RC. Mitochondria as a Therapeutic Target for Common Pathologies. Nat Rev Drug Discovery (2018) 17(12):865–86. doi: 10.1038/nrd.2018.174 30393373

[41] Luo Y Ma J Lu W. The Significance of Mitochondrial Dysfunction in Cancer. Int J Mol Sci (2020) 21(16):5598. doi: 10.3390/ijms21165598 PMC7460667 32764295

[42] Dong L Gopalan V Holland O Neuzil J. Mitocans Revisited: Mitochondrial Targeting as Efficient Anti-Cancer Therapy. Int J Mol Sci (2020) 21(21)7941. doi: 10.3390/ijms21217941 PMC7663685 33114695

[43] Zielonka J Joseph J Sikora A Hardy M Ouari O Vasquez-Vivar J. Mitochondria-Targeted Triphenylphosphonium-Based Compounds: Syntheses, Mechanisms of Action, and Therapeutic and Diagnostic Applications. Chem Rev (2017) 117(15):10043–120. doi: 10.1021/acs.chemrev.7b00042 PMC5611849 28654243

[44] Jeena MT Kim S Jin S Ryu JH. Recent Progress in Mitochondria-Targeted Drug and Drug-Free Agents for Cancer Therapy. Cancers (Basel) (2019) 12(1):4. doi: 10.3390/cancers12010004 PMC7016936 31861339

[45] Li Q Huang Y. Mitochondrial Targeted Strategies and Theirapplication for Cancer and Other Diseases Treatment. J Pharm Invest (2020) 50(3):271–93. doi: 10.1007/s40005-020-00481-0

[46] Zorova LD Popkov VA Plotnikov EY Silachev DN Pevzner IB Jankauskas SS. Mitochondrial Membrane Potential. Anal Biochem (2018) 552:50–9. doi: 10.1016/j.ab.2017.07.009 PMC5792320 28711444

[47] Dhanasekaran A Kotamraju S Karunakaran C Kalivendi SV Thomas S Joseph J. Mitochondria Superoxide Dismutase Mimetic Inhibits Peroxide-Induced Oxidative Damage and Apoptosis: Role of Mitochondrial Superoxide. Free Radical Biol Med (2005) 39(5):567–83. doi: 10.1016/j.freeradbiomed.2005.04.016 16085176

[48] Murphy MP Smith RA. Targeting Antioxidants to Mitochondria by Conjugation to Lipophilic Cations. Annu Rev Pharmacol Toxicol (2007) 47:629–56. doi: 10.1146/annurev.pharmtox.47.120505.105110 17014364

[49] Cheng G Zielonka J Dranka BP McAllister D Mackinnon AC Jr. Joseph J. Mitochondria-Targeted Drugs Synergize With 2-Deoxyglucose to Trigger Breast Cancer Cell Death. Cancer Res (2012) 72(10):2634–44. doi: 10.1158/0008-5472.can-11-3928 PMC3700358 22431711

[50] Cheng G Zielonka J McAllister DM Mackinnon AC Jr. Joseph J Dwinell MB. Mitochondria-Targeted Vitamin E Analogs Inhibit Breast Cancer Cell Energy Metabolism and Promote Cell Death. BMC Cancer (2013) 13:285. doi: 10.1186/1471-2407-13-285 23764021 PMC3686663

[51] Molina JR Sun Y Protopopova M Gera S Bandi M Bristow C. An Inhibitor of Oxidative Phosphorylation Exploits Cancer Vulnerability. Nat Med (2018) 24(7):1036–46. doi: 10.1038/s41591-018-0052-4 29892070

[52] Pan J Lee Y Cheng G Zielonka J Zhang Q Bajzikova M. Mitochondria-Targeted Honokiol Confers a Striking Inhibitory Effect on Lung Cancer *via* Inhibiting Complex I Activity. iScience (2018) 3:192–207. doi: 10.1016/j.isci.2018.04.013 30428319 PMC6137433

[53] Boyle KA Van Wickle J Hill RB Marchese A Kalyanaraman B Dwinell MB. Mitochondria-Targeted Drugs Stimulate Mitophagy and Abrogate Colon Cancer Cell Proliferation. J Biol Chem (2018) 293(38):14891–904. doi: 10.1074/jbc.RA117.001469 PMC6153299 30087121

[54] Kalyanaraman B Cheng G Zielonka J Bennett B. Low-Temperature EPR Spectroscopy as a Probe-Free Technique for Monitoring Oxidants Formed in Tumor Cells and Tissues: Implications in Drug Resistance and OXPHOS-Targeted Therapies. Cell Biochem Biophys (2019) 77(1):89–98. doi: 10.1007/s12013-018-0858-1 30259334 PMC6430651

[55] Cheng G Zielonka M Dranka B Kumar SN Myers CR Bennett B. Detection of Mitochondria-Generated Reactive Oxygen Species in Cells Using Multiple Probes and Methods: Potentials, Pitfalls, and the Future. J Biol Chem (2018) 293(26):10363–80. doi: 10.1074/jbc.RA118.003044 PMC6028982 29739855

[56] Cheng G Hardy M Topchyan P Zander R Volberding P Cui W. Potent Inhibition of Tumour Cell Proliferation and Immunoregulatory Function by Mitochondria-Targeted Atovaquone. Sci Rep (2020) 10(1):17872. doi: 10.1038/s41598-020-74808-0 33087770 PMC7578061

[57] Weinberg SE Singer BD Steinert EM Martinez CA Mehta MM Martinez-Reyes I. Mitochondrial Complex III Is Essential for Suppressive Function of Regulatory T Cells. Nature (2019) 565(7740):495–9. doi: 10.1038/s41586-018-0846-z PMC6345596 30626970

[58] Benej M Hong X Vibhute S Scott S Wu J Graves E. Papaverine and its Derivatives Radiosensitize Solid Tumors by Inhibiting Mitochondrial Metabolism. Proc Natl Acad Sci USA (2018) 115(42):10756–61. doi: 10.1073/pnas.1808945115 PMC6196495 30201710

[59] Thomlinson RH Gray LH. The Histological Structure of Some Human Lung Cancers and the Possible Implications for Radiotherapy. Br J Cancer (1955) 9(4):539–49. doi: 10.1038/bjc.1955.55 PMC2073776 13304213

[60] Brown JM Giaccia AJ. The Unique Physiology of Solid Tumors: Opportunities (and Problems) for Cancer Therapy. Cancer Res (1998) 58(7):1408–16. 9537241

[61] Brown JM. The Hypoxic Cell: A Target for Selective Cancer Therapy–Eighteenth Bruce F. Cain Memorial Award Lecture. Cancer Res (1999) 59(23):5863–70. 10606224

[62] Rockwell S Dobrucki IT Kim EY Marrison ST Vu VT. Hypoxia and Radiation Therapy: Past History, Ongoing Research, and Future Promise. Curr Mol Med (2009) 9(4):442–58. doi: 10.2174/156652409788167087 PMC2752413 19519402

[63] Ashton TM Fokas E Kunz-Schughart LA Folkes LK Anbalagan S Huether M. The Anti-Malarial Atovaquone Increases Radiosensitivity by Alleviating Tumour Hypoxia. Nat Commun (2016) 7:12308. doi: 10.1038/ncomms12308 27453292 PMC4962491

[64] Cheng G Zielonka J Ouari O Lopez M McAllister D Boyle K. Mitochondria-Targeted Analogues of Metformin Exhibit Enhanced Antiproliferative and Radiosensitizing Effects in Pancreatic Cancer Cells. Cancer Res (2016) 76(13):3904–15. doi: 10.1158/0008-5472.Can-15-2534 PMC4930686 27216187

[65] Skwarski M McGowan DR Belcher E Di Chiara F Stavroulias D McCole M. Mitochondrial Inhibitor Atovaquone Increases Tumor Oxygenation and Inhibits Hypoxic Gene Expression in Patients With Non-Small Cell Lung Cancer. Clin Cancer Res (2021) 27(9):2459–69. doi: 10.1158/1078-0432.Ccr-20-4128 PMC7611473 33597271

[66] University of Oxford. Pre-Operative Window of Opportunity Study of the Effects of Atovaquone on Hypoxia in Non-Small Cell Lung Carcinoma [Online]. University of Oxford (2018). Available at: https://clinicaltrials.gov/ct2/show/NCT02628080.

[67] Morandi A Indraccolo S. Linking Metabolic Reprogramming to Therapy Resistance in Cancer. Biochim Biophys Acta Rev Cancer (2017) 1868(1):1–6. doi: 10.1016/j.bbcan.2016.12.004 28065746

[68] Lee S Lee JS Seo J Lee SH Kang JH Song J. Targeting Mitochondrial Oxidative Phosphorylation Abrogated Irinotecan Resistance in NSCLC. Sci Rep (2018) 8(1):15707. doi: 10.1038/s41598-018-33667-6 30356107 PMC6200737

[69] Zaal EA Berkers CR. The Influence of Metabolism on Drug Response in Cancer. Front Oncol (2018) 8:500. doi: 10.3389/fonc.2018.00500 30456204 PMC6230982

[70] Hirpara J Eu JQ Tan JKM Wong AL Clement MV Kong LR. Metabolic Reprogramming of Oncogene-Addicted Cancer Cells to OXPHOS as a Mechanism of Drug Resistance. Redox Biol (2019) 25:101076. doi: 10.1016/j.redox.2018.101076 30642723 PMC6859574

[71] Zhang L Yao Y Zhang S Liu Y Guo H Ahmed M. Metabolic Reprogramming Toward Oxidative Phosphorylation Identifies a Therapeutic Target for Mantle Cell Lymphoma. Sci Transl Med (2019) 11(491):eaau1167. doi: 10.1126/scitranslmed.aau1167 31068440

[72] M.D. Anderson Cancer Center. IACS-010759 in Advanced Cancers [Online]. National Cancer Institute (NCI) (2020). Available at: https://clinicaltrials.gov/ct2/show/NCT03291938.

[73] Wang JX Choi SYC Niu X Kang N Xue H Killam J. Lactic Acid and an Acidic Tumor Microenvironment Suppress Anticancer Immunity. Int J Mol Sci (2020) 21(21)8363. doi: 10.3390/ijms21218363 PMC7664620 33171818

[74] Fares CM Allen EMV Drake CG Allison JP Hu-Lieskovan S. Mechanisms of Resistance to Immune Checkpoint Blockade: Why Does Checkpoint Inhibitor Immunotherapy Not Work for All Patients? American Society of Clinical Oncology Educational Book (2019) 39:147–64. doi: 10.1200/EDBK_240837 31099674

[75] Anderson NM Simon MC. The Tumor Microenvironment. Curr Biol (2020) 30(16):R921–r925. doi: 10.1016/j.cub.2020.06.081 32810447 PMC8194051

[76] Hanahan D Weinberg RA. The Hallmarks of Cancer. Cell (2000) 100(1):57–70. doi: 10.1016/S0092-8674(00)81683-9 10647931

[77] Tian B Wong WY Hegmann E Gaspar K Kumar P Chao H. Production and Characterization of a Camelid Single Domain Antibody-Urease Enzyme Conjugate for the Treatment of Cancer. Bioconjug. Chem (2015) 26(6):1144–55. doi: 10.1021/acs.bioconjchem.5b00237 25938892

[78] Ibrahim-Hashim A Estrella V. Acidosis and Cancer: From Mechanism to Neutralization. Cancer Metastasis. Rev (2019) 38(1-2):149–55. doi: 10.1007/s10555-019-09787-4 PMC6625834 30806853

[79] Doherty JR Cleveland JL. Targeting Lactate Metabolism for Cancer Therapeutics. J Clin Invest (2013) 123(9):3685–92. doi: 10.1172/jci69741 PMC3754272 23999443

[80] Baltazar F Afonso J Costa M Granja S. Lactate Beyond a Waste Metabolite: Metabolic Affairs and Signaling in Malignancy. Front Oncol (2020) 10:231. doi: 10.3389/fonc.2020.00231 32257942 PMC7093491

[81] Watson MJ Vignali PDA Mullett SJ Overacre-Delgoffe AE Peralta RM Grebinoski S. Metabolic Support of Tumour-Infiltrating Regulatory T Cells by Lactic Acid. Nature (2021) 591(7851):645–51. doi: 10.1038/s41586-020-03045-2 PMC7990682 33589820

[82] Mu X Shi W Xu Y Xu C Zhao T Geng B. Tumor-Derived Lactate Induces M2 Macrophage Polarization *via* the Activation of the ERK/STAT3 Signaling Pathway in Breast Cancer. Cell Cycle (2018) 17(4):428–38. doi: 10.1080/15384101.2018.1444305 PMC5927648 29468929

[83] Certo M Tsai CH Pucino V Ho PC Mauro C. Lactate Modulation of Immune Responses in Inflammatory Versus Tumour Microenvironments. Nat Rev Immunol (2021) 21(3):151–61. doi: 10.1038/s41577-020-0406-2 32839570

[84] Hardie DG Ashford ML. AMPK: Regulating Energy Balance at the Cellular and Whole Body Levels. Physiol (Bethesda) (2014) 29(2):99–107. doi: 10.1152/physiol.00050.2013 PMC3949207 24583766

[85] Carling D. AMPK Signalling in Health and Disease. Curr Opin Cell Biol (2017) 45:31–7. doi: 10.1016/j.ceb.2017.01.005 28232179

[86] Cheng G Zhang Q Pan J Lee Y Ouari O Hardy M. Targeting Lonidamine to Mitochondria Mitigates Lung Tumorigenesis and Brain Metastasis. Nat Commun (2019) 10(1):2205. doi: 10.1038/s41467-019-10042-1 31101821 PMC6525201

[87] Cheng G Hardy M Zielonka J Weh KM Zielonka M Boyle K. Mitochondria-Targeted Magnolol Inhibits OXPHOS, Proliferation, and Tumor Growth *via* Modulation of Energetics and Autophagy in Melanoma Cells. Cancer Res Treat Commun (2020) 25:100210. doi: 10.1016/j.ctarc.2020.100210 PMC7883397 32987287

[88] Harada Y Ishii I Hatake K Kasahara T. Pyrvinium Pamoate Inhibits Proliferation of Myeloma/Erythroleukemia Cells by Suppressing Mitochondrial Respiratory Complex I and STAT3. Cancer Lett (2012) 319(1):83–8. doi: 10.1016/j.canlet.2011.12.034 22210382

[89] Salminen A Kaarniranta K Kauppinen A. Phytochemicals Inhibit the Immunosuppressive Functions of Myeloid-Derived Suppressor Cells (MDSC): Impact on Cancer and Age-Related Chronic Inflammatory Disorders. Int Immunopharmacol. (2018) 61:231–40. doi: 10.1016/j.intimp.2018.06.005 29894862

[90] Trillo-Tinoco J Sierra RA Mohamed E Cao Y de Mingo-Pulido Á. Gilvary DL. AMPK Alpha-1 Intrinsically Regulates the Function and Differentiation of Tumor Myeloid-Derived Suppressor Cells. Cancer Res (2019) 79(19):5034–47. doi: 10.1158/0008-5472.Can-19-0880 PMC6774829 31409640

[91] Zeng D Long H Zhu B. Antitumor Effects of Targeting Myeloid-Derived Suppressive Cells. Trans Cancer Res (2020) 9(9):5787–97. doi: 10.21037/tcr.2020.01.52 PMC8798346 35117939

[92] Ostrand-Rosenberg S. Myeloid-Derived Suppressor Cells: Facilitators of Cancer and Obesity-Induced Cancer. Annu Rev Cancer Biol (2021) 5(1):17–38. doi: 10.1146/annurev-cancerbio-042120-105240

[93] Kim SH Li M Trousil S Zhang Y Pasca di Magliano M Swanson KD. Phenformin Inhibits Myeloid-Derived Suppressor Cells and Enhances the Anti-Tumor Activity of PD-1 Blockade in Melanoma. J Invest Dermatol (2017) 137(8):1740–8. doi: 10.1016/j.jid.2017.03.033 28433543

[94] Bu LL Yu GT Deng WW Mao L Liu JF Ma SR. Targeting STAT3 Signaling Reduces Immunosuppressive Myeloid Cells in Head and Neck Squamous Cell Carcinoma. Oncoimmunology (2016) 5(5):e1130206. doi: 10.1080/2162402x.2015.1130206 27467947 PMC4910719

[95] Trovato R Fiore A Sartori S Canè S Giugno R Cascione L. Immunosuppression by Monocytic Myeloid-Derived Suppressor Cells in Patients With Pancreatic Ductal Carcinoma is Orchestrated by STAT3. J Immunother Cancer (2019) 7(1):255. doi: 10.1186/s40425-019-0734-6 31533831 PMC6751612

[96] Caino MC Ghosh JC Chae YC Vaira V Rivadeneira DB Faversani A. PI3K Therapy Reprograms Mitochondrial Trafficking to Fuel Tumor Cell Invasion. Proc Natl Acad Sci USA (2015) 112(28):8638–43. doi: 10.1073/pnas.1500722112 PMC4507184 26124089

[97] Fischer GM Jalali A Kircher DA Lee WC McQuade JL Haydu LE. Molecular Profiling Reveals Unique Immune and Metabolic Features of Melanoma Brain Metastases. Cancer Discovery (2019) 9(5):628–45. doi: 10.1158/2159-8290.Cd-18-1489 PMC6497554 30787016

[98] Rohlenova K Sachaphibulkij K Stursa J Bezawork-Geleta A Blecha J Endaya B. Selective Disruption of Respiratory Supercomplexes as a New Strategy to Suppress Her2(high) Breast Cancer. Antioxid. Redox Signal (2017) 26(2):84–103. doi: 10.1089/ars.2016.6677 27392540 PMC5206771

[99] Chandel NS. Mitochondria as Signaling Organelles. BMC Biol (2014) 12:34. doi: 10.1186/1741-7007-12-34 24884669 PMC4035690

[100] Yu M Nguyen ND Huang Y Lin D Fujimoto TN Molkentine JM. Mitochondrial Fusion Exploits a Therapeutic Vulnerability of Pancreatic Cancer. JCI Insight (2019) 5(16):e126915. doi: 10.1172/jci.insight.126915 PMC6777817 31335325

[101] Kim G Pastoriza JM Condeelis JS Sparano JA Filippou PS Karagiannis GS. The Contribution of Race to Breast Tumor Microenvironment Composition and Disease Progression. Front Oncol (2020) 10:1022. doi: 10.3389/fonc.2020.01022 32714862 PMC7344193

[102] Deshmukh SK Srivastava SK Tyagi N Ahmad A Singh AP Ghadhban AAL. Emerging Evidence for the Role of Differential Tumor Microenvironment in Breast Cancer Racial Disparity: A Closer Look at the Surroundings. Carcinogenesis (2017) 38(8):757–65. doi: 10.1093/carcin/bgx037 PMC5862302 28430867

[103] Yao S Cheng TD Elkhanany A Yan L Omilian A Abrams SI. Breast Tumor Microenvironment in Black Women: A Distinct Signature of CD8+ T-Cell Exhaustion. J Natl Cancer Inst. (2021) 113(8):1036–43. doi: 10.1093/jnci/djaa215 PMC8328978 33395700

[104] Beebe-Dimmer JL Cooney KA. Mitochondrial Alterations may Underlie Race-Specific Differences in Cancer Risk and Outcome. J Clin Invest (2019) 129(6):2187–8. doi: 10.1172/jci128707 PMC6546466 31063989

[105] Piyarathna DWB Balasubramanian A Arnold JM Lloyd SM Karanam B Castro P. ERR1 and PGC1α Associated Mitochondrial Alterations Correlate With Pan-Cancer Disparity in African Americans. J Clin Invest (2019) 129(6):2351–6. doi: 10.1172/jci127579 PMC6546480 30920960

[106] Williams LK Padhukasahasram B Ahmedani BK Peterson EL Wells KE González Burchard E. Differing Effects of Metformin on Glycemic Control by Race-Ethnicity. J Clin Endocrinol Metab (2014) 99(9):3160–8. doi: 10.1210/jc.2014-1539 PMC4154100 24921653

